# The Impact of Article Length on the Number of Future Citations: A Bibliometric Analysis of General Medicine Journals

**DOI:** 10.1371/journal.pone.0049476

**Published:** 2013-02-06

**Authors:** Matthew E. Falagas, Angeliki Zarkali, Drosos E. Karageorgopoulos, Vangelis Bardakas, Michael N. Mavros

**Affiliations:** 1 Alfa Institute of Biomedical Sciences (AIBS), Marousi, Athens, Greece; 2 Department of Medicine and Infectious Diseases, Mitera General Hospital, Hygeia Group, Athens, Greece; 3 Department of Medicine, Tufts University School of Medicine, Boston, Massachusetts, United States of America; 4 Department of Medicine, Hygeia Hospital, Marousi, Athens, Greece; 5 Hellenic Center of Disease Control and Prevention, Marousi, Athens, Greece; 6 Department of Applied Mathematical and Physical Science, National Technical University of Athens, Athens, Greece; Aalto University, Finland

## Abstract

**Background:**

The number of citations received is considered an index of study quality and impact. We aimed to examine the factors associated with the number of citations of published articles, focusing on the article length.

**Methods:**

Original human studies published in the first trimester of 2006 in 5 major General Medicine journals were analyzed with regard to the number of authors and of author-affiliated institutions, title and abstract word count, article length (number of print pages), number of bibliographic references, study design, and 2006 journal impact factor (JIF). A multiple linear regression model was employed to identify the variables independently associated with the number of article citations received through January 2012.

**Results:**

On univariate analysis the JIF, number of authors, article length, study design (interventional/observational and prospective/retrospective), title and abstract word count, number of author-affiliated institutions, and number of references were all associated with the number of citations received. On multivariate analysis with the logarithm of citations as the dependent variable, only article length [regression coefficient: 14.64 (95% confidence intervals: (5.76–23.50)] and JIF [3.37 (1.80–4.948)] independently predicted the number of citations. The variance of citations explained by these parameters was 51.2%.

**Conclusion:**

In a sample of articles published in major General Medicine journals, in addition to journal impact factors, article length and number of authors independently predicted the number of citations. This may reflect a higher complexity level and quality of longer and multi-authored studies.

## Introduction

An article's citations are considered a measure of the scientific recognition the study has received, and thus an indicator of its value and impact on the scientific field [1]. The citations are also the main factor determining the scientific impact of a journal, as expressed by the journal impact factor [2]. This indicator represents the mean number of citations received in an index calendar year, by all the citable articles published in a journal during the previous two years [3], [4]. Researchers commonly aim to publish articles that will attract citations and will thus be regarded to have a high scientific impact, as this may be associated with their career advancement.

Several studies have been conducted to explore the factors associated with the citation count of scientific articles. While the effect of journal impact factor [5]–[10] and study design [11]–[16] on citations received has been established by different studies, the published evidence on other potentially relevant variables, such as open access to the full text of the article, [17]–[20] or article length, [13] seems conflicting.

In this context, we aimed to examine the factors associated with the number of citations received by published articles, focusing on the article's length.

## Methods

### Data sources

Original human research articles published in the first trimester of 2006 in the 5 highest impact factor journals in the field of general and internal medicine were analyzed (the *New England Journal of Medicine*, the *Lancet*, the *Journal of the American Medical Association*, the *Annals of Internal Medicine*, and the *British Medical Journal*). Experimental studies, review articles, and meta-analyses were excluded. The 2006 journal impact factors were retrieved from the Thomson Reuters Journal Citation Reports. The number of citations to each article was last assessed in January 2012, according to the Thomson Reuters Web of Knowledge.

### Data extraction

The abstract and/or full-text manuscript of each article was accessed to collect information regarding article length and characteristics that were reported to affect the number of citations in previous studies. Specifically, we documented variables comprised the number of authors and affiliated institutions, title and abstract word count, article length (as the number of pages), number of bibliographic references, study design (human or experimental studies; prospective or retrospective; interventional or observational), access to the article (open access or requiring subscription), and 2006 journal impact factor (JIF).

**Figure 1:**
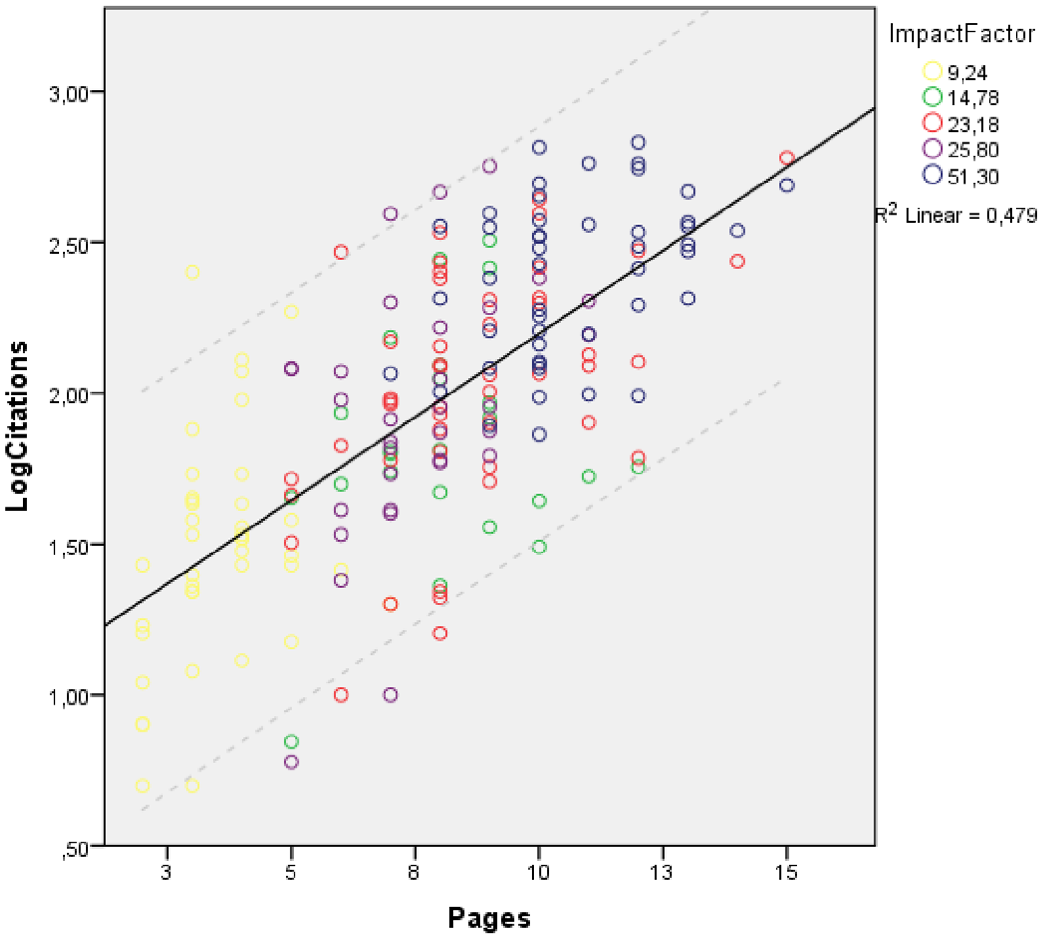
Correlation between the article length (number of print pages) and future article citations. Results from the multivariate regression analysis. The dots represent the individual pairs for the X–Y variables; the straight line is the linear regression line; the dotted lines represent the 95% confidence interval for the regression line. The different journals with their individual IF are shown in different colours.

### Data analysis and statistical methods

Statistical analyses were performed using SPSS Version 20.0. Initially, the association of each independent variable with the dependent variable (citation count) was assessed with univariate analyses (Mann-Whitney for categorical and Spearman's correlation for continuous variables); we used non-parametric methods, because citations of articles published in General Medicine journals are known to have a non-parametric distribution [21]. Variables significantly associated with the citation count in univariate analysis (p<0.10) were then entered in a backward multiple linear regression model to identify independent predictors of higher number of citations. The multiple linear regression model was also run with logarithmic transformation of the dependent variable (number of citations) to assess for a logarithmic, rather than linear relationship between the dependent and independent variables. Since the logarithmic transformed model performed better, only the results of this model were presented. To exclude the possibility of a false positive association between the article length and the number of authors and the number of citations, we repeated the multiple regression analysis separately for each of the journals, as the journal impact factor has been well established to be a major factor affecting citations.

All assumptions of linear regression were met by this model, including lack of error term correlation (Durbin-Watson = 2.013). Graphical examination of residuals did not suggest a violation of the linearity and normality assumption. Multicollinearity was deemed not important (VIF <5) for every independent variable. Homoscedasticity was checked by examination of the scatterplot of residuals and predicted values, and was met when outliers were excluded from the model. We also tested for outliers using added value and residual plots. Three outliers were identified with citations 1314, 1185 and 793, and were excluded. A variable was considered statistically significant if it had a p-value <0.05 in the final multivariable model.

## Results

A total of 196 articles were analyzed. Experimental studies were excluded, leading to a total of 192 articles. The citation count varied from 5 to 1314 with a median of 96.5 (mean = 166). The majority of studies were prospective (67.2%), open-access (90.2%) and multi-center (67.2%). The most common type of study in our sample was that of a trial (39.6%, both randomized control trials and non-randomized trials). The study characteristics are presented in Table 1.

**Table 1:** Characteristics of the analyzed studies.

Study characteristics			Hypothesis (increase citations)
No of authors, median (range)		9.88 (1–48)	More authors
No of author–affiliated institutions, median (range)		5.33 (1–43)	More institutions
Title word count, median (range)		13.75 (6–29)	Longer title
Abstract word count, median (range)		294.88 (105–589)	Longer abstract
Article length [print pages], median (range)		7.88 (2–15)	Lengthier article
No of bibliographic references, median (range)		29.31 (3–61)	More references
Nature of study	Prospective vs Retrospective	67.2% vs 30.2%	IF Prospective study
Study design	Interventional vs Observational	39.6% vs 61.4%	If Interventional study
Open access versus restricted access		90.2% vs 9.8%	If Open access
2006 journal impact factor (JIF), median (range)		26.88 (9.25–51.3)	Higher JIF
Citation count, median (range)		166.2 (5–1314)	

On univariate analysis, all tested independent variables except access (free versus restricted) and multicenter or single-center study, were found to have a statistically significant correlation to citations (Table 2). Therefore, the following variables were entered in the multivariate model: JIF, number of authors, article length, prospective or retrospective design, type of study (interventional or observational), abstract and title word count, number of affiliated institutions, and number of references, with the logarithm of the number of citations as the dependent variable.

**Table 2:** Results of statistical analysis.

Variable	Univariate		Multivariate	
	Correlation coefficient	p-value*	Regression Coefficient (95% Confidence Interval)	p-value
No of Authors	0.50	<0.001	0.003 (−0.007, 0.014)	0.555
No of author–affiliated institutions	0.35	<0.001	0.006 (−0.001, 0.014)	0.103
Title word count	−0.26	<0.001	−0.03 (−0.016, 0.009)	0.587
Abstract word count	−0.22	0.003	0.001 (0.000, 0.002)	0.107
Article length [number of print pages]	0.70	<0.001	0.079 (0.055, 0.102)	<0.001
No of References	0.33	<0.001	0.003 (−0.002, 0.008)	0.284
Retrospective study	-	<0.001	−0.045 (−0.158, 0.069)	0.438
Observational study	-	<0.001	−0.020 (−0.146, 0.107)	0.758
Multi-center study	-	0.066	Not included	-
Open access	-	0.704	Not included	-
2006 journal impact factor	0.63	<0.001	0.008 (0.004, 0.013)	<0.001

* Refers to Spearman's correlation or to Mann-Whitney U test.

**Figure 2:**
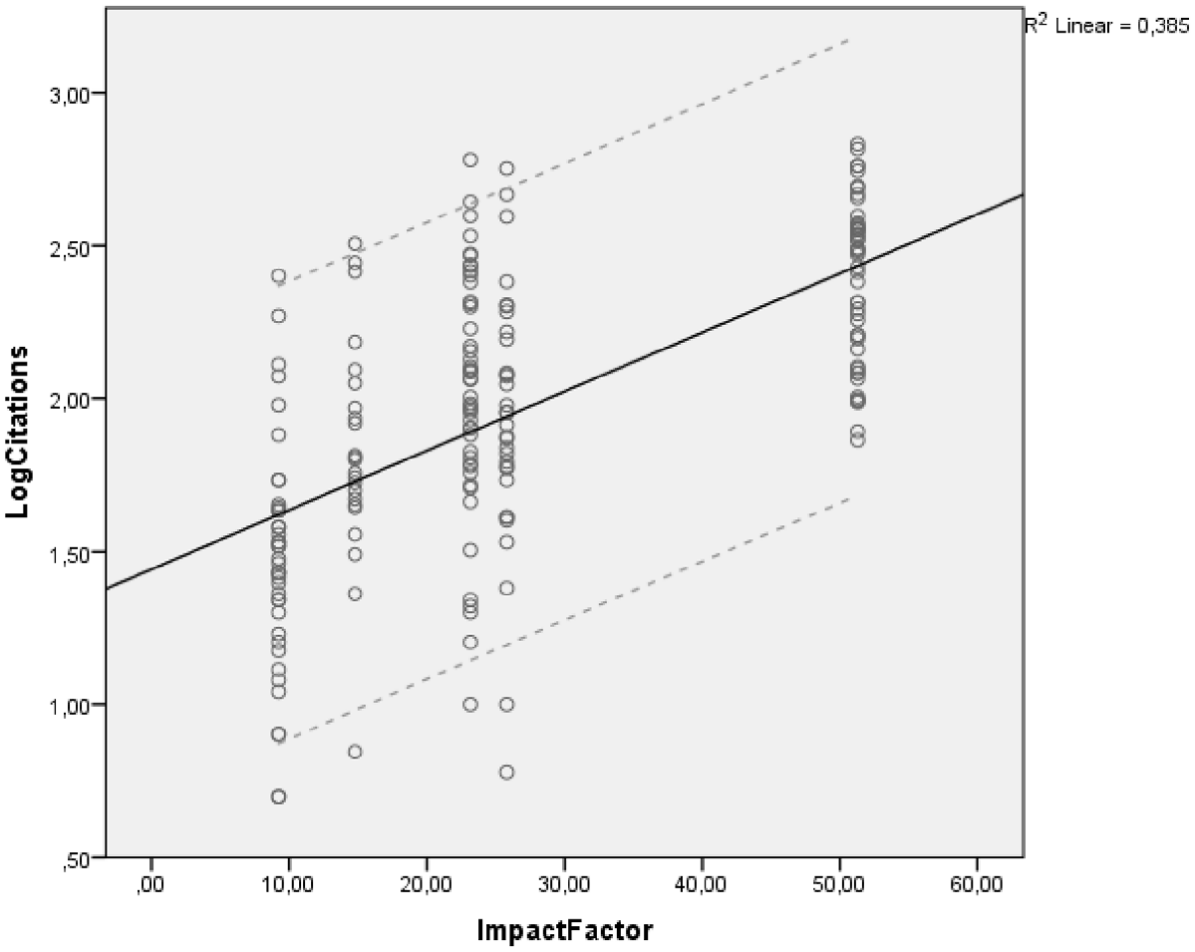
Correlation between the impact factor of the journal of publication and future article citations. Results from the multivariate regression analysis. The dots represent the individual pairs for the X–Y variables; the straight line is the linear regression line; the dotted lines represent the 95% confidence interval for the regression line.

A backward linear regression analysis was performed, removing insignificant independent variables one by one. Two variables were found to independently predict the number of citations: article length (number of pages) [regression coefficient (95% confidence interval): 0.079 (0.055–0.102), p<0.001; Figure 1and JIF [0.008 (0.004–0.013), p<0.001; Figure 2]. The variance of citations explained by these factors is 51.2% (adjusted R^2^ = 50.7%), p<0.001. The findings of the univariate and multivariate analyses are presented in table 2.

### Subgroup analyses

For the subgroup of articles published in two of the five included journals, article length was found to be the only factor independently associated with citations, with a parameter estimate of 0.080 [(0.032–0.127), p = 0.002] and 0.058 [(0.013–0.104), p = 0.013], respectively. For articles published in the third journal, statistically significant factors included the number of institutions [0.050 (0.001–0.098), p = 0.04] and the number of references [0.014 (0.003–0.024), p = 0.01], while in the fourth journal significant were the number of authors [0.029 (0.006–0.051), p = 0.015] and the number of references [0.025 (0.011–0.040), p = 0.001)]. In the remaining journal, no variable was found to be significantly associated with citations, although that may reflect the smaller sample size (n = 23). Last, article length was significantly associated with the number of citations in the singe-center studies subgroup [0.109 (0.075–0.143), p<0.001].

## Discussion

The main finding of this study is that the article length and journal impact factor are independently associated with the number of citations received by each article. Although several previous studies have reported that the journal impact factor is associated with the article citations, this is the first study, to the best of our knowledge, to report a positive association between the article length and the article citations after adjustment for several potentially confounding variables, such as the study design, prospective or retrospective nature of the study, abstract and title word count, number of author–affiliated institutions and number of bibliographic references. Specifically, we found an increase by an average of 0.079 in the logarithm of citations per article for each additional page, 0.008 for every unit of increase in the journal impact factor. The greater article length could reflect increased greater scientific complexity and higher methodological quality of a study; in addition, lengthier articles are expected to contain more information, thus increasing the possibilities that part of it will be appropriate to be cited by other researchers. Furthermore, in lengthier compared with shorter articles, the study methodology and findings could be more clearly and elaborately presented and discussed, and can therefore have a greater impact. It should be highlighted that our findings probably do not apply to long articles where the results have been improperly “inflated”; after all, some of the greatest discoveries in science have been described only briefly [22].

A few studies have assessed, albeit not comprehensively, the impact of the article length on future citations. In the field of Astronomy and Astrophysics, lengthier articles were cited more often in some journals [23]. In the fields of Infectious Diseases, Clinical Microbiology and Antimicrobial Agents, brief reports were cited less often than full articles, even after adjustment for the journal impact factor [24]. This was not the case in another study assessing 504 articles and adjusting for several confounding factors [13]. In contrast to our study, in which we assessed only original study articles, the authors included in their analysis numerous Cochrane reviews and reports from the Technology Assessment database (n = 108), that are typically lengthy; in addition, they excluded articles not meeting specific methodological and clinical relevance criteria. That study reported a slightly negative correlation between the article length and the number of citations received [−0.11 (−0.02 to −0.01)]; however, when Cochrane reviews and reports from the Technology Assessment database were excluded, no association between the article length and citations was identified. Although the difference between these findings and those of our study is probably attributed to the difference in the type of articles assessed (inclusion/exclusion of review articles), it remains to be proven whether our findings can be generalized to a larger part of the biomedical literature than just the 5 highest impact factor journals in General & Internal Medicine.

In addition to the number of print pages, we found that the impact factor of the journal and the number of authors were associated with the citation count. Although we limited our analysis only to articles from high impact factor journals, the articles published in the highest impact factor journals were cited significantly more often. It should be noted that we used the 2006 journal impact factor (that refers to articles published in 2004 and 2005) for our analysis (that referred to articles published in 2006) to avoid a potential bias. In this regard, our findings are in concordance with previous studies that found the journal impact factor to be a major predictor of the article citation count [5]–[10].

Several other variables assessed in previous studies were incorporated in our analysis, but failed to show a statistically significant association with the number of citations. The characteristics and findings of all relevant studies are briefly presented in Table 3. Some authors have described an association between the type of the study and the future citations, with more citations received by meta-analyses and randomized control trials and less citations received by observational studies [11], [12], [14], [16]; their findings are have been limited by selection bias (articles of a specific specialty) [11], [12], [14], [16] and inappropriate adjustment of confounding factors [16]. Such findings were not verified in our analysis, as we found no citation advantage neither for interventional over observational studies, nor for any specific type of study (trial, cohort, cross-sectional or case-control); however, this could also be attributed to the relatively small sample size of each subset of articles of different study type. It has been debated whether open access distribution of articles leads to more citations [18]–[20], [25], [26] or that scientific collaboration positively influence citation count [13], [15], [27]; we did not confirm such an association. Last, we did not observe a significant impact of the title length (word count) on the future citations, in contrast to what other researchers have found [25], [26]. This may be attributed to the lack of adjustment for confounding factors by those studies.

**Table 3:** Published studies examining factors that affect citations.

Author;	Years;	Analysis	Conclusions
Year	Databases/journals studied;	Dependent variable;	
	Sample size;	Independent variables	
	(Specialty)		
Main factor studied: Journal's Impact Factor (JIF)			
Perneger [5];	NR; Pubmed, Scopus	Citation comparison of consensus articles in different journals	1.0 log unit of citations increase per unit of JIF (95% CI: 0.7–1.3, P = 0.001)
2010	4 consensus statements	Citations	
	33 articles	JIF	
	(NR)		
Etter [6];	NR	Univariate and multivariate linear regression	More citations if: Statistically significant results (median 541 vs. 17, P = 0.001), higher JIF (10.2 citations/JIF point, P = 0.001)
2009	Cochrane	Citations	
	150 RCTs	JIF, Favorable outcome, Year Funding, Country, Product type	
	(Nicotine replacement RCTs)		
Filion [7];	1998–2004	NR	Journal and country most strongly associated with citations
2008	ISI Web of Science	Citation rate	(high impact factor, US)
	72 articles	Authors, JIF, Topic, Institution, Country, Year	
	(Epidemiology articles on child injuries and coronary disease)		
Nieminen [8];	1996	Mann-Whitney tests, Kruskal-Wallis, ANOVA, and negative binomial regression	No citation advantage for reporting quality and statistical analysis; JIF as important as quality
2006	Am J Psych, Arch Gen Psych, BJPsych and NJPsych	Citations	
	448 articles	Reporting quality, Sample size, JIF	
	(Psychiatry)		
Montori [9];*	2000	Multiple linear regression	Twice as many citations for systematic vs narrative reviews (95% CI: 1.5–2.7). JIF = weaker predictor than quality
2003	Hand search of 170 journals	Citations	
	271 reviews	JIF, Type of review	
	(NR)		
Callaham [10];	1991	Multivariate regression	JIF = the strongest predictor (100%); Newsworthiness score (89.9% as strong); Subjective quality score (61.5%). Positive outcome bias not significant.
2002	Emergency medicine specialty meeting articles	Citations	
	204 articles	JIF, Subject, Quality, Study design, Positive result, Newsworthiness	
	(Emergency medicine)		
Main factor studied: Study design			
Okike [11];	2002–2003	Multiple linear regression, log-transformation	More citations at 5 years in: high level of evidence, large sample size, multiple institutions, self-reported conflict of interest, sports medicine and arthroscopy. Less citations for: Pediatric orthopedic articles
2011	JBJS Am vol., JBJS Brit vol., CORR;	Citations	
	661 articles	JIF, Level of evidence, Sample size, Self-reported conflict of interest, Subject, Location, Control/blinding, No of authors/institutions, Prospective study	
	(Orthopedics)		
Willis [12];	2004	Binary logistic regression	More citation rates: RCTs [OR = 115.5 (9.4–1419.6, p<0.001)], topic of oncology [OR = 2.5 (1.4–4.7, p 0.004)]
2011	Journal of Urology, Urology, BJU, European Urology	Citation rate	
	200 articles	Study Design, Journal, Topic, Country, Sample Size, No of authors/institutions, Funding	
	(Urology)		
Lokker [13];	2005	Multiple regression	More citations if: More authors, higher clinical relevance scores, more references
2008	105 journals	Citation counts at two years	
	1274 articles	Article length, No of authors, Location, Abstract, Subject	
	(NR)		
Kulkarni [14];	1999–2000	Univariate and multivariate linear regression	Increased citation rates: larger sample, journal of publication, funding and industry-favoring result [25.7 (8.5–42.8)], cardiovascular medicine [13.3 (3.9–22.3)], oncology [12.6 (1.2–24.0)], group authorship [11.1 (2.7–19.5)]
2007	Lancet, JAMA, NEJM	Annual rate of citations	
	328 articles	Industry funding, Industry-favoring result, Clinical category of article, Group authorship, JIF, Sample size	
	(NR)		
Bhandari [15];	2000	Regression analysis	Citations: Meta-analyses (mean = 15.5), Randomized trials (9.3), Basic science papers (7.6), Observational (retrospective 5.3, prospective 4.2), Case reports (1.5)
2007	The JBJS Am vol.	Citations	
	137 original articles	Study design, Sample size, Location, Topic	
	(Orthopedics)		
Patsopoulos [16];	1991, 2001	Logistic regression	Significantly more citations for meta-analyses
2005	NR	Citations in 2 years	
	2646 articles	Year, Country, JIF, Design	
	(NR)		
Main factor studied: Open access (OA)			
Kim [17];	2009–2010	NR	43% decrease in citations per month from 2009 to 2010 (p = 0.00064). No difference in 2009 vs 2010 simulated
2011	Journal of American Medical Informatics Association	Citations per month comparison between 2009 (paid) and 2010 (OA) articles	
	NR	Paid versus open access	
	(NR)		
Lansingh [18];	2003	Univariate general linear model	No statistical significance of OA. Significant factors included No of authors, country, subject, language, and funding.
2009	Scopus, GoogleScholar	Citations	
	480 articles	OA, No of authors, Country, Subject, Language, Funding	
	(Opthalmology)		
Davis [19];**	2007	Logistic and negative binomial regression	OA: 89% more full text downloads (76–103%), 42% more PDF downloads (32–52%), 23% more unique visitors (16–30%), 24% less abstract downloads (− 29–19%). No evidence of citation advantage.
2008	American Psychological Society	Citations after 12 months	
	1619 articles	OA	
	(Psychology)		
Eyesenbach [20];	2004–2005	Logistic and linear regression	OA articles more recognized and cited [OR 2.1 (1.5–2.9)]
2003	PNAS	Citations	
	1492 original research articles	OA, No of authors, Country, Funding, Authors' lifetime publication count	
	(NR)		
Main factor studied: No of authors			
Figg [25];	(1975–1985) and 1995	Multiple logistic and linear regression analysis	More citations as No of authors increases
2006	Science, Cell, Nature, NEJM, Lancet, JAMA	Citations	
	9,415 articles	No of authors and institutions	
	(NR)		
Main factor studied: Title length			
Hazibzadeh [26];*	2005	Linear regression model	Increased citation rates in longer titles (more in high JIF)
2010	22 English journals via Scopus	Citations	
	9031 articles	Title length	
	(NR)		
Jacques [27];	2005	NR	Increased citation rates in: Longer titles (rho = 0.62, 2-sided P<0.0001), presence of a colon or acronym in title
2010	Lancet, BMJ, Journal of Clinical Pathology	Citations	
	50 articles	Title characteristics	
	(NR)		
Main factor studied: Hit count online			
Perneger [28];	1999	NA	More citations for papers with most hits on BMJ website the first week: (extra 3.7 citations/100 hits, P<0.001)
2004	BMJ		
	153 articles		
	(NR)		

All studies were cohort studies of published articles except: * Cross-sectional studies, ** Randomized control trial.

Abbreviations: Am J Psych: American Journal of Psychiatry, Arch Gen Psych: Archives of General Psychiatry, BJPsych: British Journal of Psychiatry, BMJ: British Medical Journal, CORR: Clinical Orthopedics and Related Research, JAMA: Journal of American Medical Association, JBJS Am vol.: Journal of Bone and Joint Surgery American Volume, JBJS Brit vol.: Journal of Bone and Joint Surgery British Volume JIF: Journal's Impact factor, NEJM: New England Journal of Medicine, NJPsych: Nordic Journal of Psychiatry, OA: Open Access, OR: Odds ratio, PNAS: Proceedings of the National Academy of Sciences, RCT: Randomized control trial.

Our study is subject to certain limitations. First, it is characterized by selection bias, as the articles published in high impact factor journals in General Medicine may not be representative of all published articles; for example, they are more likely to be multi-center RCT than a single-center case-control study. Second, although our results are statistically significant, it is possible that the association does not represent a causal relationship. Third, we did not assess the analyzed articles regarding topic [11], [15], [16], paper quality [9], [10], funding [15], [18] or country of origin of the authors [7], [18], which are factors that have been found to affect citations by other authors. Last, in our assessment of article length, we only analyzed page count (not word count) and inter-journal variance in the number of words per page cannot be excluded.

In conclusion, for original research articles published in the major General Medicine journals, in addition to journal impact factor, the article length independently predicts the number of future citations. This probably reflects a higher complexity level and quality of longer studies and does not apply to inappropriately inflated articles. Additional studies are warranted to verify the generalizability of our findings to a largest part of the biomedical literature.

## References

[1] Cheek J, Garnham B, Quan J (2006) What's in a number? Issues in providing evidence of impact and quality of research(ers). Qual Health Res 16: 423–435. 16449691 10.1177/1049732305285701

[2] Falagas ME, Kouranos VD, Arencibia-Jorge R, Karageorgopoulos DE (2008) Comparison of SCImago journal rank indicator with journal impact factor. FASEB J 22: 2623–2628. 18408168 10.1096/fj.08-107938

[3] Falagas ME, Alexiou VG (2008) The top-ten in journal impact factor manipulation. Arch Immunol Ther Exp (Warsz) 56: 223–226. 18661263 10.1007/s00005-008-0024-5

[4] Garfield E (1996) How can impact factors be improved? BMJ 313: 411–413. 8761234 10.1136/bmj.313.7054.411 PMC2351785

[5] Perneger TV (2010) Citation analysis of identical consensus statements revealed journal-related bias. J Clin Epidemiol 63: 660–664. 20097531 10.1016/j.jclinepi.2009.09.012

[6] Etter JF, Stapleton J (2009) Citations to trials of nicotine replacement therapy were biased toward positive results and high-impact-factor journals. J Clin Epidemiol 62: 831–837. 19128941 10.1016/j.jclinepi.2008.09.015

[7] Filion KB, Pless IB (2008) Factors related to the frequency of citation of epidemiologic publications. Epidemiol Perspect Innov 5: 3. 18302781 10.1186/1742-5573-5-3 PMC2291053

[8] Nieminen P, Carpenter J, Rucker G, Schumacher M (2006) The relationship between quality of research and citation frequency. BMC Med Res Methodol 6: 42. 16948835 10.1186/1471-2288-6-42 PMC1570136

[9] Montori VM, Wilczynski NL, Morgan D, Haynes RB (2003) Systematic reviews: a cross-sectional study of location and citation counts. BMC Med 1: 2. 14633274 10.1186/1741-7015-1-2 PMC281591

[10] Callaham M, Wears RL, Weber E (2002) Journal prestige, publication bias, and other characteristics associated with citation of published studies in peer-reviewed journals. JAMA 287: 2847–2850. 12038930 10.1001/jama.287.21.2847

[11] Okike K, Kocher MS, Torpey JL, Nwachukwu BU, Mehlman CT, et al (2011) Level of evidence and conflict of interest disclosure associated with higher citation rates in orthopedics. J Clin Epidemiol 64: 331–338. 20947295 10.1016/j.jclinepi.2010.03.019

[12] Willis DL, Bahler CD, Neuberger MM, Dahm P (2011) Predictors of citations in the urological literature. BJU Int 107: 1876–1880. 21332629 10.1111/j.1464-410X.2010.10028.x

[13] Lokker C, McKibbon KA, McKinlay RJ, Wilczynski NL, Haynes RB (2008) Prediction of citation counts for clinical articles at two years using data available within three weeks of publication: retrospective cohort study. BMJ 336: 655–657. 18292132 10.1136/bmj.39482.526713.BE PMC2270947

[14] Bhandari M, Busse J, Devereaux PJ, Montori VM, Swiontkowski M, et al (2007) Factors associated with citation rates in the orthopedic literature. Can J Surg 50: 119–123. 17550715 PMC2384258

[15] Kulkarni AV, Busse JW, Shams I (2007) Characteristics associated with citation rate of the medical literature. PLoS One 2: e403. 17476325 10.1371/journal.pone.0000403 PMC1852582

[16] Patsopoulos NA, Analatos AA, Ioannidis JP (2005) Relative citation impact of various study designs in the health sciences. JAMA 293: 2362–2366. 15900006 10.1001/jama.293.19.2362

[17] Kim HE, Jiang X, Kim J, Ohno-Machado L (2011) Trends in biomedical informatics: most cited topics from recent years. J Am Med Inform Assoc 18 Suppl 1i166–170. 22180873 10.1136/amiajnl-2011-000706 PMC3241182

[18] Lansingh VC, Carter MJ (2009) Does open access in ophthalmology affect how articles are subsequently cited in research? Ophthalmology 116: 1425–1431. 19545905 10.1016/j.ophtha.2008.12.052

[19] Davis PM, Lewenstein BV, Simon DH, Booth JG, Connolly MJ (2008) Open access publishing, article downloads, and citations: randomised controlled trial. BMJ 337: a568. 18669565 10.1136/bmj.a568 PMC2492576

[20] Eysenbach G (2006) Citation advantage of open access articles. PLoS Biol 4: e157. 16683865 10.1371/journal.pbio.0040157 PMC1459247

[21] Falagas ME, Kouranos VD, Michalopoulos A, Rodopoulou SP, Batsiou MA, et al (2010) Comparison of the distribution of citations received by articles published in high, moderate, and low impact factor journals in clinical medicine. Intern Med J 40: 587–591. 20718883 10.1111/j.1445-5994.2010.02247.x

[22] Watson JD, Crick FH (1953) Molecular structure of nucleic acids: a structure for deoxyribose nucleic acid. Nature 171: 737–738. 13054692 10.1038/171737a0

[23] Ball P (2008) A longer paper gathers more citations. Nature 455: 274–275. 10.1038/455274a 18800099

[24] Mavros M, Bardakas V, Rafailidis P, Sardi T, Demetriou E, et al (2013) Comparison of number of citations to full original articles versus brief reports. Scientometrics 94(1): 203–206.

[25] Habibzadeh F, Yadollahie M (2010) Are shorter article titles more attractive for citations? Cross-sectional study of 22 scientific journals. Croat Med J 51: 165–170. 20401960 10.3325/cmj.2010.51.165 PMC2859422

[26] Jacques TS, Sebire NJ (2010) The impact of article titles on citation hits: an analysis of general and specialist medical journals. JRSM Short Rep 1: 2. 21103094 10.1258/shorts.2009.100020 PMC2984326

[27] Figg WD, Dunn L, Liewehr DJ, Steinberg SM, Thurman PW, et al (2006) Scientific collaboration results in higher citation rates of published articles. Pharmacotherapy 26: 759–767. 16716129 10.1592/phco.26.6.759

[28] Perneger TV (2004) Relation between online “hit counts” and subsequent citations: prospective study of research papers in the BMJ. BMJ 329: 546–547. 15345629 10.1136/bmj.329.7465.546 PMC516105

